# Osteoporosis Knowledge and Health Beliefs Among Female Community Leaders in Peru

**DOI:** 10.1089/whr.2019.0005

**Published:** 2020-02-07

**Authors:** Mihaela Sava, Leslie Yingzhijie Tseng, Maria Valderrama, David Mabey, Patricia J. García, Evelyn Hsieh

**Affiliations:** ^1^ Clinical Research Department, Faculty of Infectious and Tropical Diseases, London School of Hygiene and Tropical Medicine, London, United Kingdom.; ^2^ Section of Rheumatology, Allergy, and Immunology, Yale School of Medicine, New Haven, Connecticut.; ^3^ Epidemiology, STI, HIV Research Unit, School of Public Health, Universidad Peruana Cayetano Heredia, Lima, Peru.

**Keywords:** osteoporosis, osteoporosis knowledge test, Osteoporosis Health Belief Scale, Peru, community leaders

## Abstract

***Background:*** Osteoporosis presents an increasing problem globally, primarily affecting older adults and postmenopausal women. Among important modifiable risk factors for osteoporosis, physical activity and calcium intake help reduce bone mineral loss and decrease the prevalence of osteoporosis. Although osteoporosis knowledge and health beliefs are associated with adopting preventive behavior and screening rates, few studies have evaluated them in Latin American populations.

***Materials and Methods:*** We conducted a cross-sectional study among female community leaders in a Peruvian periurban setting. A total of 60 women participated in the study, with a mean age of 43.7 ± 8.3 years, mean body mass index of 30.4 ± 5.3 kg/m^2^, 88% being overweight or obese, and 58.3% having completed high school education or beyond. Participants completed translated and culturally adapted Osteoporosis Knowledge Test and Osteoporosis Health Belief Scale *via* semistructured interviews.

***Results:*** Most participants reported high knowledge regarding osteoporosis, perceived benefits to exercise and calcium intake, and health motivation. The level of osteoporosis knowledge was highly associated with the level of education, and we found a trend for the association between level of knowledge and perceived benefits of exercise and barriers to calcium intake among participants.

***Conclusions:*** Female community leaders with high health motivation and community engagement can contribute enormously to osteoporosis prevention programs in local communities in the future.

## Introduction

Osteoporosis, defined by compromised bone strength predisposing a person to an increased risk of fracture,^1^ is becoming a significant public health concern globally. One osteoporotic fracture is believed to occur every 3 seconds worldwide, causing >9 million fractures annually.^2^ Disability caused by fractures leads to high health care costs, prolonged hospital stays, and impaired mobility.^3–5^ With an estimated increase in hip fracture rates by more than threefold in the next 50 years, treatment and care of such fractures are expected to incur staggering social and economic costs.^6^

The problem is projected to be particularly severe in middle- and low-income countries,^7^ where demographic and economic transitions result in a rapidly aging population and an increasing number of city dwellers, juxtaposed with fragmented infrastructure and human resources to diagnosis and manage osteoporosis.^8^

In contrast to the abundant knowledge in high-income countries, limited data exist regarding the osteoporosis epidemiology in Latin America,^9^ and the majority of the studies have focused only on vertebral fractures.^10,11^ However, prevention and early intervention play crucial roles in combating osteoporotic fracture and result in better outcomes.^12^ Adopting preventive behaviors such as adequate calcium and vitamin D intake and regular exercise is critical in delaying the onset and progression of this disease.^13,14^ Moreover, knowledge and health beliefs regarding osteoporosis play a vital role in the adoption of osteoporosis-preventive behaviors and increasing screening rates.^15,16^

The Health Belief Model has been widely used to predict adoption of preventive behaviors associated with osteoporosis risk factors.^17^ Its evaluation is based upon seven key domains: perceived susceptibility, perceived seriousness, benefits of exercise, benefits of calcium intake, barriers to exercise, barriers to calcium intake, and health motivation.^18^ Several validated scales have been developed specifically for osteoporosis prevention, including the osteoporosis knowledge test (OKT) and Osteoporosis Health Belief Scale (OHBS).^19^

These scales have been adapted to evaluate and guide the intervention of osteoporosis preventive behaviors in many middle- and low-income countries.^20–23^ Few studies have addressed the level of osteoporosis knowledge and beliefs among nonhealth care professionals in Latin America^11,24^ and existing data suggest that osteoporosis knowledge often fails to translate into preventive behaviors among the general population.^25,26^

To better understand the level of knowledge, risk factors, and health beliefs regarding osteoporosis in Peru, we conducted an exploratory study among a group of female peer leaders in a Peruvian periurban community. Of the 32 million people living in Peru, 7% of the women between ages 40 and 60 years and ∼30% of women aged 60 years or above are estimated to suffer from osteoporosis.^27^ Interventions aimed at promoting osteoporosis-preventive behaviors in the general population do not yet exist in Peru.^24^ By evaluating osteoporosis-related knowledge and health beliefs held by female community leaders, we assessed the potential of this population to serve as promoters of bone health in future community-based osteoporosis interventions.

## Materials and Methods

### Study design and sampling

We conducted a cross-sectional study among female community leaders from a periurban setting in the district of Ventanilla, Callao, Peru. A total of 136 women with motivation to help the locals and potential interest to participate were identified from a database of a previous study examining the role of community champions in cervical cancer prevention conducted by investigators at Cayetano Heredia University.^28^

In July 2015, 71 eligible participants were contacted by the study staff 1 or 2 days before data collection to briefly explain the purpose of the study and agree on a meeting if the person was willing to participate. Among all women contacted by the study staff, 60 agreed to participate in this study. Further health education regarding osteoporosis was given to all participants at the end of the interview. This study was approved by the ethics committees of the Universidad Peruana Cayetano Heredia and the London School of Hygiene and Tropical Medicine.

### Data collection and measures

We merged two internationally validated instruments, the revised OKT and the OHBS, originally developed by Kim et al.^29^ and revised by von Hurst and Wham,^30^ into one questionnaire evaluating the level of knowledge and health beliefs among the female community leaders. Permission to use the revised OKT and the OHBS was obtained from the authors. In addition, we collected data on the sociodemographic and clinical characteristics of the participants using a general health questionnaire. Questionnaires were translated into Spanish and piloted with 12 female participants. After the pilot study, a culturally adapted version of revised OKT and the OHBS with linguistic changes and a simplified Likert's scale for the OHBS were implemented.

Data were collected in July 2015 using face-to-face interviews conducted by an experienced native Spanish speaker. Data were collected using cell phones installed with the Open Data Kit program. Each participant was interviewed privately after receiving an information sheet and giving a verbal consent. Participants were also verbally informed of the purpose, voluntary nature, anonymity, and technical instructions. No incentive payments were offered. The duration for completing one questionnaire was between 15 and 25 minutes. Further training regarding osteoporosis prevention was provided to all participants at the end of the interview that lasted ∼30 minutes.

### Survey instruments

#### Knowledge

We translated and culturally adapted the revised OKT, modified by von Hurst and Wham to include two questions on vitamin D,^30^ to measure osteoporosis knowledge among the participants. The revised OKT is a 26-item multiple-choice instrument consisting of two subscales: exercise (items 1–18) and nutrition (items 1–11 and 19–26), with 14 shared items addressing general knowledge about osteoporosis and its risk factors. The number of correct answers to the exercise-subscale questions and nutrition-subscale questions represents the exercise score (range: 0–18) and the nutrition score (range: 0–19), respectively. The total number of all correct responses gives rise to the total knowledge score (range: 0–26).

Literature suggests that internal consistency evaluated by KR20 coefficients for all three scales of the revised OKT ranges between 0.81 and 0.85, and test-retest analysis demonstrated a Pearson correlation coefficient of 0.87.^31^ For the purpose of this study, we consider participants with an exercise score <10 to have lower exercise knowledge, and those with ≥10 to have higher exercise knowledge; participants with a nutrition score <13 to have lower nutrition knowledge and those with ≥13 to have higher nutrition knowledge; and participants with a total score <16 to have lower osteoporosis knowledge and those with ≥16 to have higher osteoporosis knowledge.

#### Health beliefs

The OHBS is a 42-item tool examining health beliefs toward developing osteoporosis across seven 6-item domains: susceptibility, seriousness, benefits of exercise, benefits of calcium intake, barriers to exercise, barriers to calcium intake, and health motivation. In the original survey, participants rate each item from five options (1: strongly disagree to 5: strongly agree). A Cronbach's coefficient alpha of 0.85 for prevention intention has been reported.^32^

However, preliminary testing among 12 women suggested that the 5-point Likert's scale presented challenges in comprehension for participants. As a result, we simplified the answer options into three choices (1: disagree, 2: neutral, and 3: agree), yielding an adjusted range of each domain of 6 to 18 and total possible range of 42 to 126. For each health beliefs domain, we divided participants into two groups: lower health beliefs (score: 6–12) and higher health beliefs (score: 13–18).

#### Sociodemographic and clinical characteristics

We collected sociodemographic information regarding age (<45 or ≥45 years), education level (less than high school vs. high school education or beyond), marital status (single/divorced/separated/widowed vs. married/cohabitant).

We were also interested in the general osteoporosis risk factors of our study population, including: body mass index (BMI) (underweight, normal, or overweight/obese), smoking history (ever vs. never), current alcohol use of three or more drinks in one sitting (yes or no), history of a fall within the last year (yes or no), parental history of fracture (yes, no, or do not know), personal history of prior fracture (yes vs. no/do not know/do not remember), prior bone density test (yes vs. no/do not know/do not remember), prior diagnosis of osteoporosis (yes or no), current use of nutritional supplements (calcium, vitamin D, cod liver oil, or none of the above), use of assistive walking device (yes or no), noticed height loss of 2 cm or more (yes or no).

Finally, the survey collected reproductive health-related factors including: age at first menstrual bleeding (<12 years vs. ≥12 years), last menstrual bleeding (<12 months vs. ≥12 months ago), parity (<4 children vs. ≥4 children), age at first birth (≤18, 19–25, >25 years old or no children), prior removal of one or both ovaries (yes or no), and use of hormone replacement therapy (ever vs. never).

### Data analysis

We described the sample characteristics using standard frequency analyses, means and standard deviations, medians, and interquartile range for all variables, as appropriate. We further examined differences between participants with different levels of education using independent *t*-tests and χ^2^ tests. The outcome of each OKT and OHBS subscale was reported using descriptive statistics.

#### Multivariable regression

We adopted both unadjusted and adjusted linear regression models to examine the associations between each of our independent variables (sociodemographic and clinical characteristics and measure of osteoporosis health beliefs) and the major outcome (total osteoporosis knowledge).

We first performed univariate analysis to determine associations between the level of osteoporosis knowledge and explanatory variables with a minimum of five participants in each category. No multicollinearity was found, and correlations among the explanatory variables are all <0.40.

Two multivariant models were constructed for sociodemographic and clinical characteristics, and osteoporosis health beliefs, respectively. We fit the multivariable model using backward regression^33^ starting with variables with *p*-value <0.20 in the unadjusted analyses. Variables with the largest *p*-value in each linear regression model were removed one at a time until the model with the largest adjusted *R*^2^ was obtained. Results were reported as unadjusted and adjusted standardized beta coefficient with 95% confidence intervals. We considered statistical evidence of an association *p*-value 0.05 to be significant. All statistical analyses were performed using SPSS Statistics version 25 (IBM, Armonk, NY).

## Results

### Sociodemographic and clinical characteristics of sample

A total of 60 out of 72 potentially eligible participants enrolled in this study, yielding a participation rate of 85%. The participants had a mean age of 43.7 ± 8.3 years and a mean BMI of 30.4 ± 5.3 kg/m^2^ (Table 1). More than half of the participants completed high school education or beyond (58.3%), were married or cohabiting (71.7%), and had no more than three children (60%). A quarter of the participants (25%) gave birth to their first child at the age of 18 or younger. Participants with higher level of education had fewer children (*p* < 0.001). About 50% of the women reported height loss of 2 cm or more, and 35% of the sample were postmenopausal, neither of whom varied significantly by level of education.

**Table 1.** Sociodemographic, Clinical Characteristics, and Osteoporosis Knowledge and Beliefs, Entire Cohort and Stratified by Education Level

Variable	Entire Cohort (*N* = 60)	Level of education	
		Education<high school^a^ (*N* = 25)	Education≥high school^b^ (*N* = 35)
Sociodemographic characteristics			
Age, mean ± SD	43.7 ± 8.3	43.8 ± 7.8	43.6 ± 8.8
Marital status, *n* (%)			
Married/cohabitant	43 (71.7)	21 (84.0)	22 (62.9)
Single/divorced/separated/widowed	17 (28.3)	4 (16.0)	13 (37.1)
General osteoporosis risk factors			
Body mass index, mean ± SD, kg/m^2^	30.4 ± 5.3	31.1 ± 5.1	30 ± 5.5
Smoking, ever, *n* (%)	5 (8.3)	3 (12.0)	2 (5.7)
Current alcohol use, *n* (%)	1 (1.7)	1 (4.0)	0 (0)
History of a fall within the past year, *n* (%)	11 (18.3)	5 (20.0)	6 (17.1)
Parental history of hip fracture, *n* (%)	8 (13.3)	3 (12.0)	5 (14.3)
Personal history of fracture, *n* (%)	6 (10.0)	2 (8.0)	4 (11.4)
Prior bone density test, *n* (%)	3 (5.0)	2 (8.0)	1 (2.9)
Prior diagnosis of osteoporosis, *n (%)*	2 (3.3)	1 (4.0)	1 (2.9)
Current use of nutritional supplements for bone health, *n* (%)	5 (8.3)	3 (12.0)	2 (5.7)
Current use of an assistive device for walking, *n* (%)	0 (0)	0 (0)	0 (0)
Noticed height loss of 2 cm or more, *n* (%)	30 (50.0)	14 (56.0)	16 (45.7)
Reproductive health-related factors			
Age at first menstrual bleeding, mean ± SD	13.1 ± 1.9	13.2 ± 1.8	13.1 ± 2
Menstrual status, *n (%)*			
Premenopausal	39 (65.0)	**20 (80.0)**	19 (54.3)^*^
Postmenopausal	21 (35.0)	**5 (20.0)**	16 (45.7)^*^
Parity, *n* (%)			
0–3 children	36 (60.0)	**9 (36.0)**	27 (77.1)^***^
≥4 children	24 (40.0)	**16 (64.0)**	8 (22.9)^***^
Age at first birth, mean ± SD	21.8 ± 5	**19.7 ± 3.3**	23.4 ± 5.5^**^
Prior removal of one or both ovaries, *n* (%)	4 (6.7)	1 (4.0)	3 (8.6)
Use of hormone replacement therapy, ever, *n* (%)	2 (3.3)	1 (4.0)	1 (2.9)
OKT (revised),^c^ mean ± SD			
Exercise	11.2 ± 2.5	**10.4 ± 2.9**	11.8 ± 1.9^*^
Calcium	13 ± 2.8	**11.8 ± 3**	13.9 ± 2.3^**^
Total	16.3 ± 3	**14.8 ± 3.4**	17.3 ± 2.3^**^
Osteoporosis Health Belief Scale,^d^ mean ± SD			
Susceptibility	13 ± 3.3	12.5 ± 3.5	13.3 ± 3.3
Seriousness	13.3 ± 3.1	14.1 ± 3	12.8 ± 3
Benefits of exercise	16.6 ± 2.3	15.9 ± 2.9	17 ± 1.6
Benefits of calcium	15.4 ± 2.1	15.4 ± 2.6	15.4 ± 1.6
Barriers to exercise	9.2 ± 2.3	9.6 ± 2.4	8.9 ± 2.2
Barriers to calcium	8.6 ± 2.3	9.4 ± 2.8	8.1 ± 1.7
Health motivation	15.4 ± 2.2	15.4 ± 2.3	15.3 ± 2.1

Bold values indicate significant difference between the two groups compared.

^a^ No formal education, some primary school, completed primary school, or some secondary school.

^b^ Completed secondary school, some or completed occupational institution or university.

^c^ In the revised OKT, exercise score ranges from 0 (lowest) to 20 (highest), calcium score ranges from 0 (lowest) to 26 (highest), total knowledge score ranges from 0 (lowest) to 32 (highest).

^d^ In Osteoporosis Health Belief Scale, each subscale score ranges from 6 (disagree) to 18 (agree).

^*^ *p* ≤ 0.05.

^**^ *p* ≤ 0.01.

^***^ *p* ≤ 0.001.

SD, standard deviation; OKT, osteoporosis knowledge test.

### Osteoporosis knowledge and beliefs

The cohort received a moderately high average total knowledge score (16.3 ± 3.0 out of 32), with a lower average exercise knowledge score (11.2 ± 2.5 out of 26), and higher average calcium knowledge score (13.0 ± 2.8 out of 20) (Table 1). Among the seven OHBS domains, participants felt neutral regarding the susceptibility (13.0 ± 3.3) and seriousness (18.5 ± 4.0) of osteoporosis and reported high perceived benefits to exercise (16.6 ± 2.3) and calcium intake (15.4 ± 2.0) and health motivation (15.4 ± 2.2). At the same time, we observed low perception of barriers to exercise (9.2 ± 2.3) and barriers to dietary calcium intake (8.6 ± 2.3).

Participants with higher level of education demonstrated more osteoporosis knowledge with regard to both exercise (*p* < 0.05) and calcium (*p* < 0.01). In addition, they were slightly more likely to consider themselves susceptible to osteoporosis and perceive higher benefits to preventive behaviors, whereas their peers reported slightly higher perceived seriousness of osteoporosis and perceived barriers to assessing preventive practices (Table 1).

### Association between osteoporosis knowledge and explanatory variables

In unadjusted analysis of sociodemographic and clinical characteristics, participants with higher level of education had significantly higher osteoporosis knowledge scores (*p* < 0.01) (Table 2). In the adjusted analysis, participants with higher level of education again showed significantly higher osteoporosis knowledge scores (*p* < 0.05), with parental history of hip fracture and age at first birth contributing to the final model. In unadjusted analysis of osteoporosis health beliefs, participants reporting higher perceived benefits of calcium were also more likely to have higher osteoporosis knowledge scores (*p* < 0.05). No statistically significant results yielded from the adjusted analysis, although multiple health belief scales contributed to the final model.

**Table 2.** Unadjusted and Adjusted Linear Regression Models for Osteoporosis Knowledge

Sociodemographic and clinical characteristics	Unadjusted		Adjusted		Osteoporosis Health Belief Scale	Unadjusted		Adjusted	
	Standardized beta	95% CI	Standardized beta	95% CI		Standardized beta	95% CI	Standardized beta	95% CI
Age	−0.11	−0.13 to 0.06			Susceptibility	0.14	−0.11 to 0.36		
Married/cohabitant	−0.08	−2.3 to 1.2			Seriousness	−0.23	−0.48 to 0.03	−0.16	−0.41 to 0.09
High school education or above	**0.40**	0.94 to 3.89^**^	**0.30**	0.14 to 3.25^*^	Benefits of exercise	0.23	−0.04 to 0.64	0.21	−0.05 to 0.61
Body mass index	−0.18	−0.25 to 0.05	—	—	Benefits of calcium	0.16	−0.15 to 0.61		
Smoking, ever	−0.17	−4.62 to 1.02			Barriers to exercise	−0.14	−0.52 to 0.17	—	—
History of a fall within the past year	−0.11	−2.89 to 1.17			Barriers to calcium	**−0.27**	−0.69 to −0.02^*^	−0.24	−0.64 to 0.02
Parental history of hip fracture	0.21	−0.4 to 4.15	0.20	−0.51 to 3.92	Health motivation	−0.05	−0.43 to 0.3		
Personal history of fracture	0.16	−1.03 to 4.18							
Use of nutritional supplements for bone health	−0.15	−4.41 to 1.25							
Noticed height loss of 2 cm or more	−0.09	−2.14 to 1.01							
Age at first menstrual bleeding	−0.02	−0.44 to 0.38							
Postmenopausal	0.18	−0.48 to 2.78	—	—					
Parity	−0.23	−2.96 to 0.18	—	—					
Age at first birth	0.23	−0.02 to 0.28	0.15	−0.07 to 0.25					

Bold values indicate significant difference between the two groups compared.

^*^ *p* ≤ 0.05.

^**^ *p* ≤ 0.01.

CI, confidence interval.

## Discussion

In this study, we found that female community leaders in a periurban setting in Peru possessed a moderately high level of knowledge regarding osteoporosis and its risk factors, with high levels of health motivation and lower levels of perceived barriers toward behavioral change. Among this population, participants who completed high school education or beyond demonstrated higher levels of osteoporosis knowledge. This exploratory study is among the few studies that address the osteoporosis knowledge and beliefs among nonhealth care professionals in Latin America, and the first to explore the feasibility of mobilizing this population as key promoters to further expand the knowledge and preventive health behaviors in future community-based osteoporosis interventions.

Women participating in this study demonstrated a relatively high knowledge regarding osteoporosis in contrast to similar studies conducted among Hispanic women in the United States, El Salvador, and Brazil.^26,34–36^ This finding was not surprising, given that the participants were selected from a relatively empowered group of women, who were interested in learning about health (cervical cancer) and in helping other women to learn how to care about their health, highlighting their high level of health motivation and lower levels of perceived barriers toward behavioral change.

However, the level of knowledge may be lower in the general population of the community, as suggested by our finding that the level of education correlates with the level of knowledge regarding osteoporosis, which is consistent with prior studies from Latin America.^34,35^ Therefore, targeted osteoporosis prevention programs would have major implications in closing the gap, in preventive health behaviors in general and in osteoporosis knowledge in particular, due to the inadequacy or absence of health education received previously by women in these communities.

Furthermore, as previous studies across different cultural backgrounds have suggested, community-based and/or peer-led targeted interventions are highly effective in improving general knowledge regarding osteoporosis.^37–39^ In Peru, motivated community leaders have proved to be valuable assets in community-based health interventions,^40,41^ suggesting that our study participants could play a crucial role in extending the benefits of future osteoporosis prevention programs to more members of the community.

Although demographic characteristics such as age, education level, marital status, and menopausal status were diverse among participants, the social and clinical characteristics and risk factors for osteoporosis were very similar across this group. It can be hypothesized that these women are representative of female community leaders in periurban settings across Peru. However, more data are needed to extrapolate our findings to the general population in Lima, Peru, as well as other parts of the country or region.

Given that no prior data on osteoporosis knowledge and behavior was available from Peru, we consider being able to adapt the tool and to use it successfully as a pilot to be an important first step in expending our understanding of the general population of this region. The study design focused on female community leaders in the hope that they could serve as key population for future health promotion efforts. Although we recognize that the study participants are likely to be better educated and more readily to adopt preventative health behaviors than the general population, it is helpful to gain a baseline understanding of their osteoporosis knowledge and health beliefs for future community mobilization in prevention efforts.

One prominent characteristic of participants in our study was that the vast majority of the participants were overweight or obese, with an average BMI >30 kg/m^2^. A previous study regarding nutrition in Lima, Peru, identified lack of information about appropriate serving sizes and lack of access to healthy recipes as major barriers to achieving a healthier diet.^42^ Exposure to urban environments and migration, both common in a periurban environment, are also associated with higher odds of obesity as well.^43^

Higher BMI is traditionally perceived to act as a protective factor for osteoporosis.^44^ However, populations suffering from overweight or obesity also tend have a more sedentary lifestyle,^45^ and a sedentary lifestyle is, in turn, associated with reduced bone mass and increased risk of other comorbidities that further prevent the adoption of exercise as preventive health behaviors.^46^

In addition, although several studies have looked into child and infant nutrition status and market access in Peru,^47,48^ little is known about the nutritional status and access to dietary calcium among the general population. Micronutrient deficiencies have been associated with obesity,^49^ which could, in turn, contribute to the prevalence of vitamin D deficiency in overweight and obese populations.

Therefore, we recommend that future osteoporosis prevention programs tailored to periurban community-dwelling women in Peru should prioritize lifestyle intervention, more specifically expanding access to exercise facilities and professional guidance, promoting healthier diet patterns that include calcium-rich foods, as well as providing community-level motivation and support with the help of local community leaders.

Our study has several limitations. First, the study has a relatively small sample size of 60 participants, limiting the resolution of the effects of risk factors detected in data analysis. However, this limitation was an unfortunate necessity in an exploratory study; our primary goal was to assess the feasibility of evaluating osteoporosis knowledge and health beliefs among Peruvian women using a translated and culturally adapted version of the revised OKT and OHBS, so that larger scale studies in the future can validate the OKT and the OHBS in Spanish, as well as evaluate the efficacy of the simplified Likert scale for the OHBS.

Second, the study findings cannot be extrapolated to represent the general population in Peru or Latin America. The participants in our study were preselected female community leaders with a high social status who may distinguish themselves from the general female population at risk for osteoporosis in this region by age, higher education level, and motivation in health promotion.

Meanwhile, a second goal of this exploratory study was to identify the research questions for future studies with larger sample size and communities across different regions, given the scarcity of data on preventive behavior for bone health in Latin America. Through this study, we identified populations that could play an essential role in promoting future osteoporosis awareness and prevention campaigns with community mobilization, as supported by their osteoporosis knowledge, appreciation of the preventive behaviors, and community leadership.

Finally, due to limited time and resources we chose a cross-sectional design, and, therefore, are not able to infer causality in the relationships assessed between potential risk factors and outcomes. Nor could we measure all variables influencing the uptake of osteoporosis-preventive behaviors or barriers to osteoporosis screening. However, because exercise and dietary calcium intake are cost-efficient and modifiable behaviors that serve as cornerstones of most prevention programs, they are key informative variables in the context of resource-limited settings.

In summary, women in our study exhibited a moderately high average level of osteoporosis knowledge, which was significantly and positively associated with levels of education. These community leaders were very motivated with high appreciation for the benefits of osteoporosis preventive behaviors and perceived low barriers to adopting these behaviors. Targeted interventions to raise osteoporosis awareness in the general population should include motivated community leaders, such as the participants of this study, to encourage an early start of preventive osteoporosis behavior among young women and improved screening rates in women.

## Abbreviations Used

BMI: body mass index
CI: confidence interval
OHBS: Osteoporosis Health Belief Scale
OKT: osteoporosis knowledge test
SD: standard deviation
